# Synthesis and evaluation of coumarin derivatives against human lung cancer cell lines

**DOI:** 10.1590/1414-431X20176455

**Published:** 2017-09-12

**Authors:** K.-G. Weng, Y.-L. Yuan

**Affiliations:** 1 Department of Radiation Oncology, Chongqing Cancer Institute and Hospital and Cancer Center, Chongqing, China; 2 Department of Clinical Laboratory, Chongqing the Seventh People's Hospital, Chongqing, China

**Keywords:** Coumarin, X-ray, Lung cancer

## Abstract

Series of novel coumarin derivatives [I (a–d) and II (a–d)] were successfully synthesized and their structures were determined based on infrared 1H-nuclear magnetic resonance (NMR), HRMS, and single crystal X-ray crystallography. Additionally, the new synthesized compounds were evaluated to identify the molecular characteristics that contribute to their cytotoxicity, which was tested against SK-LU-1, SPC-A-1 and 95D human lung cancer cell lines, using the MTT assay. The results of this study showed that compounds Ic, Id, IIc, and IId exhibited an efficient percentage of inhibition of cell proliferation.

## Introduction

Cancer is the leading cause of death in developed countries and the second leading cause of death in developing countries (1–3). Currently, the traditional therapies of surgery, chemotherapy and radiation play an important role in the systemic treatment of cancer (4–6). However, the treatment outcome is generally poor. Thus, it is very important to find an effective alternative treatment for cancer (7–9).

**Figure 1.**
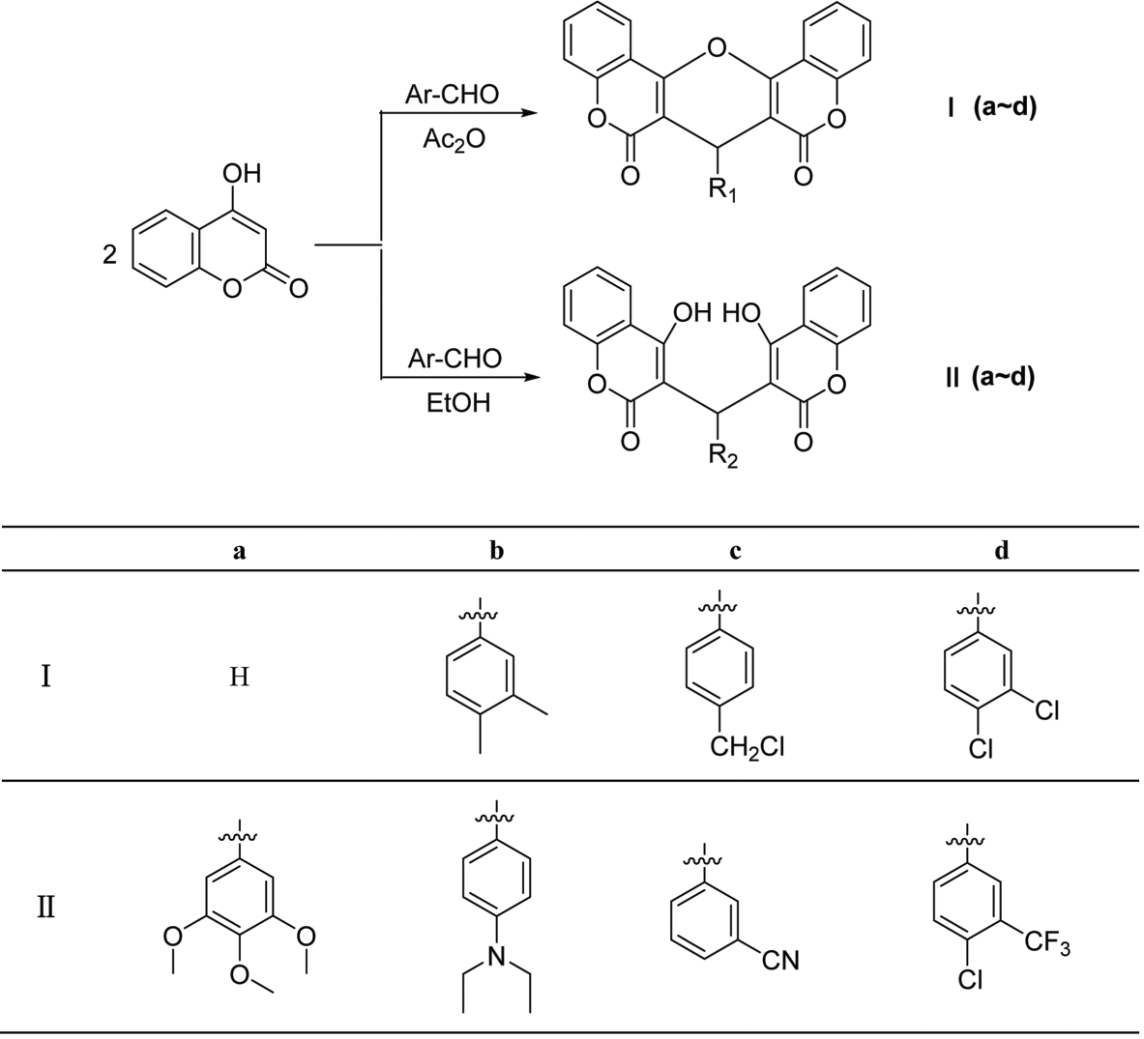
Synthesis route of compounds I (a–d) and II (a–d).

Coumarins are an important class of compounds of both natural and synthetic origin. Many compounds that contain the coumarin moiety exhibit useful and diverse pharmaceutical and biological activities, and there has been a growing interest in their synthesis (10–12). Some of these coumarin derivatives have been found useful in photochemotherapy, antitumor and anti-HIV therapy, as CNS-stimulants, antibacterial, anticoagulant, antifungal, and antioxidant agents, and as dyes (13–15). Natural, semi-synthetic and synthetic coumarins are useful substances in drug research. Coumarins can be used not only to treat cancer, but also to treat the side effects caused by radiotherapy (16–18). In this paper, series of coumarin derivatives [I (a–d) and II (a–d)] (Figure 1) have been synthesized and their potential antitumor activity was investigated.

## Material and Methods

### Apparatus and materials

To obtain infrared (IR) spectra (400–4000 cm^−1^), we used a Brucker Equinox-55 spectrophotometer (Bruker, Germany), to obtain 1H-nuclear magnetic resonance (NMR) spectra we used a Varian Inova-400 spectrometer (Virian USA; at 400 MHz), and for the Mass spectra, we used a micrOTOF-Q II mass spectrometer (Bruker, Germany). The melting points were taken on a XT-4 micro melting apparatus (Ledon, China), and the thermometer was uncorrected.

### Synthesis and characterization of compounds I (a–d) and II (a–d)

Compounds I (a–d) were synthesized according to the methods of a previous report (19). A mixture of aromatic aldehydes (10 mmol) and 4-hydroxycoumarin (20 mmol) was dissolved in 100 mL of acetic anhydride (Ac2O). A few drops of piperidine were added, and the mixture was stirred for 3 h at room temperature. After reaction completion, determined by thin-layer chromatography, water was added until precipitation occurred. After filtering the precipitates, they were sequentially washed with ice-cooled water and ethanol and then dried in a vacuum.

*I a*. 7H-pyrano[3,2-c;5,6-c′]dichromene-6,8-dione: 279–280°C. IR (KBr pellet cm^–1^): 3432, 2844, 1943, 1564, 1108 cm^–1^. 1H NMR (DMSO-d6, δ, ppm): 7.912–7.960 (m, 2H), 7.562–7.647 (m, 2H), 7.321–7.389 (m, 4H), 3.789 (s, 1H), 3.680 (s, 1H). HRMS (ESI+): m/z: calcd for C19H10O5: 341.0420 [M+Na+]; found: 341.0419.

*I b*. 7-(3,4-dimethyl-phenyl)-7H-pyrano[3,2-c;5,6-c′]dichromene-6,8-dione: >300°C. IR (KBr pellet cm^–1^): 3433, 2834, 1870, 1581, 1060 cm^–1^. 1H NMR (DMSO-d6, δ, ppm): 8.388–8.407(d, 2H), 7.747–7.765(t, 2H), 7.497–7.567(m, 4H), 7.127(s, 1H), 7.066–7.085(d, 1H), 7.009–7.029(d, 1H), 4.803(s, 1H), 2.127–2.137(d, 6H). HRMS (ESI+): m/z: calcd for C27H18O5: 445.1046 [M+Na+]; found: 445.1098.

*I c*. 7-(4-chloromethyl-phenyl)-7H-pyrano[3,2-c;5,6-c′]dichromene-6,8-dione: >300°C. IR (KBr pellet cm^–1^): 3390, 2731, 1771, 1479, 1075 cm^–1^. 1H NMR (DMSO-d6, δ, ppm): 8.405–8.428 (q, 2H), 7.757–7.800 (m, 2H), 7.506–7.578 (m, 4H), 7.403–7.424 (d, 2H), 7.329–7.350 (d, 2H), 4.899 (s, 1H), 4.701 (s, 2H). HRMS (ESI+): m/z: calcd for C26H15ClO5: 465.0500 [M+Na+]; found: 465.0564.

*I d*. 7-(3,4-dichloro-phenyl)-7H-pyrano[3,2-c;5,6-c′]dichromene-6,8-dione: >300°C. IR (KBr pellet cm^–1^): 3329, 2853, 1799, 1521, 1120 cm^–1^. 1H NMR (DMSO-d6, δ, ppm): 8.398–8.421 (q, 2H), 7.753–7.806 (m, 3H), 7.501–7.578 (m, 6H), 4.901 (s, 1H). HRMS (ESI+): m/z: calcd for C25H12Cl2O5: 484.9954 [M+Na+]; found: 484.9919.

The synthesis method for compounds II (a–d) was similar to that of compounds I (a–d), in which Ac2O solvent was replaced by ethanol.

*II a*. 3,3′-((3,4,5-trimethoxyphenyl)methylene)bis(4-hydroxy-2H-chromen-2-one): 211–212°C. IR (KBr pellet cm^–1^): 3066, 2672, 1912, 1654, 987 cm^–1^. 1H NMR (CDCl3, δ, ppm): 11.586(s, 1H), 11.310(s, 1H), 8.048–8.092(d, 2H), 7.641–7.684(m, 2H), 7.431–7.452(d, 4H), 6.441–6.443(d, 2H), 6.097(s, 1H), 3.873(s, 3H), 3.742(s, 6H). HRMS (ESI+): m/z: calcd for C28H22O9: 525.1156 [M+Na+]; found: 525.1177.

*II b*. 3,3′-((4-(diethylamino)phenyl)methylene)bis(4-hydroxy-2H-chromen-2-one): 221–222°C. IR (KBr pellet cm^–1^): 3411, 2732, 1878, 1675, 1219 cm^–1^. 1H NMR (CDCl3, δ, ppm): 8.033–8.061(m, 2H), 7.615–7.651(t, 2H), 7.379–7.432(q, 4H), 7.039–7.060(d, 2H), 6.634–6.655(d, 2H), 6.048(s, 1H), 3.324–3.376(q, 4H), 1.145–1.180(t, 6H). HRMS (ESI+): m/z: calcd for C29H25NO6: 506.1574 [M+Na+]; found: 506.1519.

*II c*. 3-(bis(4-hydroxy-2-oxo-2H-chromen-3-yl)methyl)benzonitrile: 242–243°C. IR (KBr pellet cm^–1^): 3422, 2754, 1910, 1678, 1029 cm^–1^. 1H NMR (CDCl3, δ, ppm): 11.564(s, 1H), 11.353(s, 1H), 7.996–8.094(q, 2H), 7.649–7.688(t, 2H), 7.575–7.593(t, 1H), 7.390–7.507(m, 7H), 6.069(s, 1H). HRMS (ESI+): m/z: calcd for C26H15NO6: 460.0792 [M+Na+]; found: 460.0766.

*II d*. 3,3′-((4-chloro-3-(trifluoromethyl)phenyl)methylene)bis(4-hydroxy-2H-chromen-2-one): 219–220°C. IR (KBr pellet cm^–1^): 3011, 2875, 2000, 1765, 1101 cm^–1^. 1H NMR (CDCl3, δ, ppm): 11.568 (s, 1H), 11.384 (s, 1H), 8.017–8.121 (q, 2H), 7.668–7.707 (t, 2H), 7.355–7.519 (m, 7H), 6.079 (s, 1H). HRMS (ESI+): m/z: calcd for C26H14ClF3O6: 537.0323 [M+Na+]; found: 537.0398.

### Crystal structure determination

According to the evaporation of chloroform solution, suitable single crystals of compound I b became available. The diffraction data were acquired on a Bruker Smart Apex CCD area detector using a graphite monochromated Mo Kα radiation (λ=0.71073 Å) at room temperature. The structure was solved by using the program SHELXL-97 (20) and Fourier difference techniques, and refined by full-matrix least-squares method on F2. Hydrogen atoms attached to carbon were placed in geometrically idealized positions and refined using a riding model. Crystallographic data for compound I b are reported in Table 1.

**Table 1.** Crystal data and structure refinements for compound I b.

Formula	C27H18O5·CHCl3
Mr	541.78
Temperature/K	293 (2)
Crystal system	Monoclinic
Space group	P21/c
a/Å	15.1120 (16)
b/Å	11.8560 (12)
c/Å	14.6020 (14)
α/°	90
β/°	111.633 (6)
γ/°	90
V/Å3	2431.9 (4)
Z	4
Dcalc/g·cm^-3^	1.480
μ(Mo Kα)/mm^−1^	3.745
θ range/°	3.15 to 72.74
Reflections collected	26067
No. unique data [R(int)]	4754 [0.0625]
No. data with I ≥ 2σ(I)	4278
R1	0.0487
ωR2 (all data)	0.1182

### Cytotoxicity assay

The MTT (3-(4,5-dimethylthiazol-2-yl)-2,5-diphenyl-2H-tetrazolium bromide) assay was performed as described previously (21). Cells were seeded onto a 96-well plate at a concentration of 10^4^ cells/well and allowed to adhere overnight. Five replicates were prepared for each treatment and cultured for 48 or 72 h. After 20 μL of MTT (5 mg/mL) was added to each well, the cells were cultured for another 4 h. The supernatant was discarded. After 150 μL of DMSO was added to each well, the samples were incubated at 37°C for 30 min and then swirled for 10 min. The absorbance at 570 nm was measured using a microplate reader. Experiments were repeated three times.

## Results and Discussion

### Molecular structure

**Figure 2.**
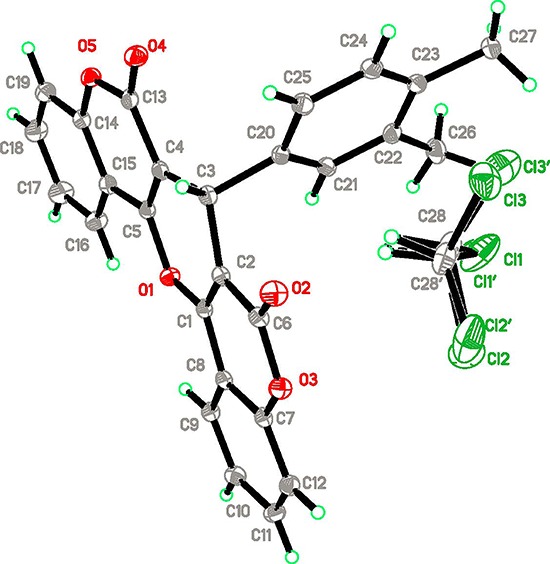
Molecular structure of compound I b.

In order to confirm the configuration of the product, single crystals of compound I b were cultured for X-ray diffraction analysis. The crystal structure of compound I b is given in Figure 2. One can see a chloroform solvent molecule incorporated in the asymmetric unit. In general, the molecular dimensions of the title compound agree with the corresponding values of other similar compounds (9). In the title crystal structure, two 4-hydroxycoumarin moieties are linked through a methylene bridge, wherein one hydrogen atom has been replaced with a 3,4-dimethylphenyl residue. Two hydroxyl groups form an ether bond by releasing a water molecule. This results in the formation of a big, essential planar heterocyclic ring system with 4.349° and 5.217° dihedral angle between the newly formed ether ring and the two original coumarin rings, respectively. The dihedral angle between the mean planes of the heterocyclic ring system and the 3,4-dimethylphenyl ring is 87.219°.

**Figure 3.**
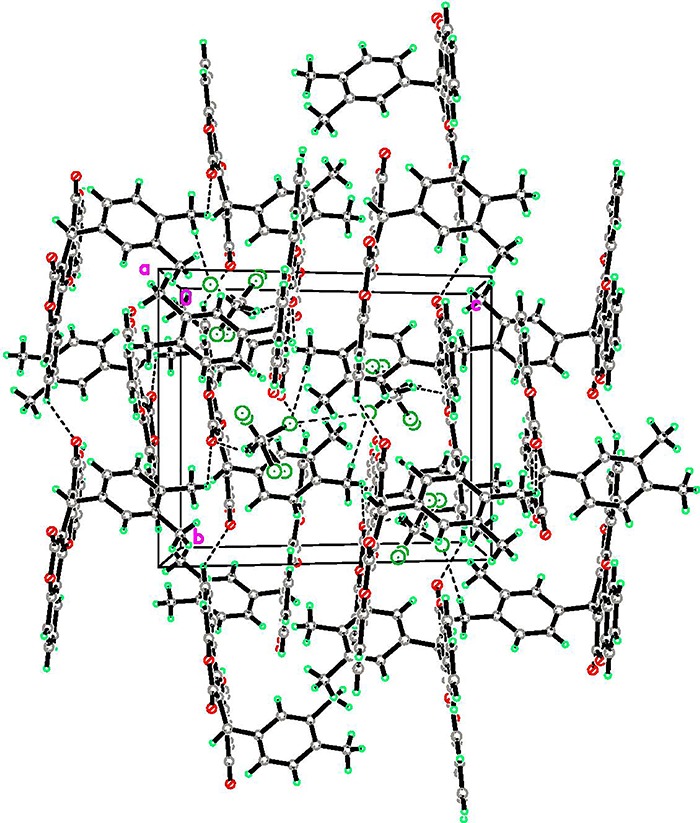
Crystal packing structure of compound I b along the a axis.

The crystal packing structure along the a axis is shown in Figure 3. Its crystal structure represents a three-dimensional supra-molecular network. Within the three-dimensional structure, there is one type of C–H…O; a hydrogen bonding is observed among these organic ligands. Detailed information of C–H…O hydrogen bonding is reported in Table 2. In other words, the three-dimensional supra-molecular framework was generated by means of the C–H…O hydrogen bonding interactions.

**Table 2.** Hydrogen bonds information (Angstrom, Deg).

D–H…A	D–H	H…A	D…A	D-H…A	Symmetry code
C11–H11…O4	0.9500	2.5400	3.1714	124.00	x, 1+y, z
C27–H27B…O2	0.9800	2.5300	3.143	121.00	x, 1/2-y, 1/2+z
C28–H28…O2	1.0000	2.1700	3.079	150.00	

### Pharmacology

The synthesized compounds I (a–d) and II (a–d) were screened *in vitro* for their antitumor activities against SK-LU-1, SPC-A-1 and 95D human lung cancer cell lines using the standard MTT method, with the antitumor drug docetaxel used as a positive control. The *in vitro* cytotoxicity screening assays were conducted at different compound concentrations. All of the experiments were carried out in triplicate. The IC50 values were calculated from the percentage of cytotoxicity by nonlinear curve fitting and are presented in Table 3.

**Table 3.** *In vitro* anti-proliferative activity of I (a–d) and II (a–d) against human cancer cell lines.

Compounds	IC50 (μM)		
	SK-LU-1	SPC-A-1	95D
I a	>100	>100	>100
I b	>100	>100	>100
I c	20	20	23
I d	25	25	21
II a	>100	>100	>100
II b	>100	>100	>100
II c	20	21	25
II d	22	20	23
Doxorubicin	25	30	25

Among all of the investigated compounds, I c, I d, II c and II d exhibited the most potent growth inhibition in the three lung cancer cells, with an IC50 value of 20–25 μM, indicating that they are more potent than docetaxel, which exhibited an IC50 value of 25–30 μM. In contrast, other compounds exhibited significantly weaker activity, because the cytotoxicity was lower than that of docetaxel in the three cell lines, with an IC50 value of >100 μM.

In conclusion, among the coumarin compounds that we successfully synthesized, compounds I c, I d, II c and II d had a strong electron-withdrawing group (-CH2Cl, -Cl, -CN and -CF3) in the phenyl ring with the highest cytotoxic activity against the cancer cell lines tested. Therefore, the four compounds are potential anticancer drugs, yet further tests are required to determine their *in vivo* antitumor activity.
